# An optomechanogram for assessment of the structural and mechanical properties of tissues

**DOI:** 10.1038/s41598-020-79602-6

**Published:** 2021-01-11

**Authors:** W. Lee, A. Ostadi Moghaddam, S. Shen, H. Phillips, B. L. McFarlin, A. J. Wagoner Johnson, K. C. Toussaint

**Affiliations:** 1 Department of Mechanical Engineering, University of Colorado Boulder, Boulder, CO 80309 USA; 2 Department of Mechanical Science and Engineering, University of Illinois at Urbana-Champaign, Champaign, IL 61820 USA; 3 Center for Health, Aging, and Disability (CHAD), College of Applied Health Science, University of Illinois at Urbana-Champaign, Champaign, IL 61820 USA; 4 Department of Veterinary Clinical Medicine, University of Illinois at Urbana-Champaign, Urbana, IL 61801 USA; 5 Department of Women, Children and Family Health Science, University of Illinois College of Nursing, Chicago, IL 60612 USA; 6 Carle Illinois College of Medicine, University of Illinois at Urbana-Champaign, Champaign, IL 61820 USA; 7 Carl R. Woese Institute for Genomic Biology, University of Illinois at Urbana-Champaign, Urbana, IL 61801 USA; 8 School of Engineering, Brown University, Providence, RI 02912 USA

**Keywords:** Multiphoton microscopy, Tissue engineering, Biomedical engineering, Mechanical engineering

## Abstract

The structural and mechanical properties of tissue and the interplay between them play a critical role in tissue function. We introduce the optomechanogram, a combined quantitative and qualitative visualization of spatially co-registered measurements of the microstructural and micromechanical properties of any tissue. Our approach relies on the co-registration of two independent platforms, second-harmonic generation (SHG) microscopy for quantitative assessment of 3D collagen-fiber microstructural organization, and nanoindentation (NI) for local micromechanical properties. We experimentally validate our method by applying to uterine cervix tissue, which exhibits structural and mechanical complexity. We find statistically significant agreement between the micromechanical and microstructural data, and confirm that the distinct tissue regions are distinguishable using either the SHG or NI measurements. Our method could potentially be used for research in pregnancy maintenance, mechanobiological studies of tissues and their constitutive modeling and more generally for the optomechanical metrology of materials.

## Introduction

In mammals, the four tissue types—connective, muscular, epithelial, and neural—each have specific functions such as providing the framework for the body’s organs, facilitating movement, forming a protective barrier, and regulating signals internal and external to the body, respectively. The composition and spatial arrangement of the tissue constituents, i.e., its structure, dictate the tissue’s mechanical behavior and biological function. A range of diseases are known to cause changes in tissue structure and mechanical behavior, such as tendinosis^1, 2^, ligament injuries^3^, osteoporosis^4^, liver fibrosis^5^, and aortic valve stenosis^6^, and these can arise from congenital defects or from acute or chronic injury. Therefore, it is important to understand the structure and mechanical function of healthy, diseased, abnormal, and damaged tissue as this understanding can help to diagnose and treat the underlying cause.

The characterization of tissue structural and mechanical properties, and the relationship between them, is experimentally challenging for a number of reasons. Many tissues are spatially heterogeneous, hierarchical, and elastically anisotropic. Typical mechanical testing techniques, like tension and compression tests, do not capture the heterogeneity of the tissue and the characterization of the local anisotropy is challenging. Often, direct visual inspection of a tissue’s structure is achieved through the use of optical (brightfield) microscopy and exogenous stains, e.g., hematoxylin and eosin (H&E). This approach, while very successful, is time consuming and limited to 2D inspection. Thus, no information about 3D tissue structural heterogeneity can be directly obtained. These limitations are especially restrictive for complex tissues, like those that are spatially and temporally heterogeneous and anisotropic, whereby the nature of the relative contributions to overall bulk mechanical properties from distinct spatial regions is not well understood. Other advanced imaging technologies and mechanical property testing methods such as second-harmonic generation (SHG) microscopy and nanoindentation (NI) may help to overcome these challenges by offering new solutions for assessing the 3D tissue microstructure and micromechanical properties at sub-micron scales.

A variety of optical methods have been used to assess the microstructure of collagen tissue because of its primary load bearing role. Standard fluorescence microscopy has been used to investigate the use of fluorescence in collagen tissue as a biomarker for differentiating normal and dysplastic tissues^7^. As another approach, researchers used commercial spectral-domain optical coherence tomography (OCT) to analyze the tissue structure–function relationship of complex collagen structures^8^. Lastly, small angle light scattering^9^ and polarized light microscopy^10^ have been used to estimate collagen fiber alignment. These methods have shown that the alignment of collagen fibers is closely related to the applied strain and tissue stiffness. Notwithstanding the importance of these studies, the out-of-plane fibers in the tissue are not considered due to the technical limitations of acquiring 3D images and the complexity of the data analysis. SHG microscopy has emerged as an attractive alternative for imaging collagen fibers. Unlike the aforementioned imaging methods, SHG microscopy is based on nonlinear optical scattering, whereby intense optical pulses interacting with a medium with a 2nd-order optical nonlinearity produce much weaker light at half the wavelength (or twice the frequency) of the original^11^. Biological structures such as fibrillar collagen yield strong SHG signals, making the technique attractive for noninvasive and label-free biological imaging in three spatial dimensions. Furthermore, owing to the technique’s specificity to structure, content in SHG images can be quantified using relatively accessible image processing methods. Indeed, this type of quantitative SHG (qSHG) imaging based on structural characterization has attracted increased interest as a reliable method for quantitative assessment of the collagen network in a variety of tissues including tendon, bone, ligament, and rat cervix^2, 3, 12, 13^.

An increasingly accepted method for characterizing the mechanical properties of soft tissues like brain^14^, cartilage^15^, and cornea^16^, as examples, is NI. This technique can quantify the local elastic, viscoelastic, and poroelastic mechanical properties of soft materials by indenting a microscale, geometrically well-defined probe into the sample and measuring the resulting load and indentation depth. The load-indentation data are processed using a well-established elastic contact theory^17^. Equilibrium properties, such as elastic and shear moduli, permeability and diffusivity, and time-dependent properties, such as creep and stress relaxation response, can be determined at precise locations, allowing for tissue properties to be mapped in-plane with micron-scale resolution. NI is particularly well suited to capture the heterogeneity of the mechanical properties of tissues. While the structural and mechanical properties of the tissue have been extensively studied independently, the direct link between the local microstructural and micromechanical properties has not been investigated. This gap limits our understanding of the microstructure-mechanical properties relationship of the tissue and the potential clinical solutions it can provide. Measuring and co-registering the properties using NI and SHG microscopy can fill in this knowledge gap. Characterization of tissues with complex collagen microstructure, such as cervix, provides a good platform for developing and testing this new methodology.

The cervix is an organ between the uterus and vagina that has not been extensively studied. The structure and function of cervical tissue is of interest because of its important biomechanical role during pregnancy^18^; it should stay firm to maintain the intrauterine position of the fetus, but must undergo dramatic changes as pregnancy progresses^19^. The cervical extracellular matrix (ECM) is made of a heterogeneous network of collagen fibers, with fibers arranged both in- and out-of-plane, and glycosaminoglycans (GAGs), elastin, and water^20^. The collagen fiber microstructure dramatically changes during pregnancy^21^, making collagen organization not only spatially, but also temporally heterogeneous. Some research has shown that the structural changes during cervical remodeling occur nonuniformly across the cervix cross-section^22^. To visualize and quantify the collagen structure of cervix, researchers have used SHG microscopy to study cervix tissue due to the aforementioned advantages. SHG microscopy studies have quantitatively measured parameters such as mean intensity, forward-to-backward signal intensity ratio, collagen fiber size and porosity to assess cervical remodeling in mice^23^, and SHG has been adapted to endoscopic technologies—demonstrating strong clinical potential^24^. SHG microscopy has also been applied to human cervix to determine the collagen fiber alignment^25^ and identify correlations with ultrasound imaging^26^. By using the optical sectioning capability, SHG imaging has been previously used to analyze the 3D collagen structure of a few tissues, such as articular cartilage^27^, but the 3D SHG images have not been quantitatively analyzed to distinguish different regions of cervix, where the collagen structure is complex. To characterize the elastic and viscoelastic properties of human cervical tissue, a number of studies used standard indentation and inverse finite element analysis (e.g., ^28^), but they were limited to millimeter-scale indentation. Besides assessment, co-registration of both the 3D collagen microstructure and accompanying mechanical properties of the cervix, while less studied, can help to elucidate their roles in overall biological function. Understanding cervical structure–function has significant potential to help predict and treat spontaneous preterm birth (sPTB)^29^.

Even with the significance of the aforementioned studies, methods that can extract 3D structure and mechanical properties from the same region in a tissue microstructure do not currently exist. In this paper, we use NI and qSHG microscopy to report the spatially heterogeneous mechanical properties and 3D microstructure of cervical tissue, respectively. NI measures the heterogeneous indentation modulus at the microscale, and qSHG distinguishes distinct cervical regions according to the 3D microstructural parameters. In addition to these independent measurements, we employ the combined approaches of NI and qSHG microscopy to demonstrate co-registered measurements of cervix stiffness and 3D collagen-fiber organization at the micrometer scale. We find that the structural and mechanical properties are region-specific and the indentation modulus measured from NI is strongly correlated with qSHG parameters related to collagen concentration and dispersion of collagen-fiber orientation in 2D and 3D. We introduce the optomechanogram to visualize the correlations between the collagen fiber organization and the mechanical properties. Our findings can contribute to developing more accurate methods for evaluating the risk of sPTB by explaining the effect of tissue microstructure on the mechanical behavior of the cervix, but are also quite broadly applicable to any heterogeneous collagenous tissue.

## Results

### SHG imaging of non-pregnant rat cervices

**Figure 1:**
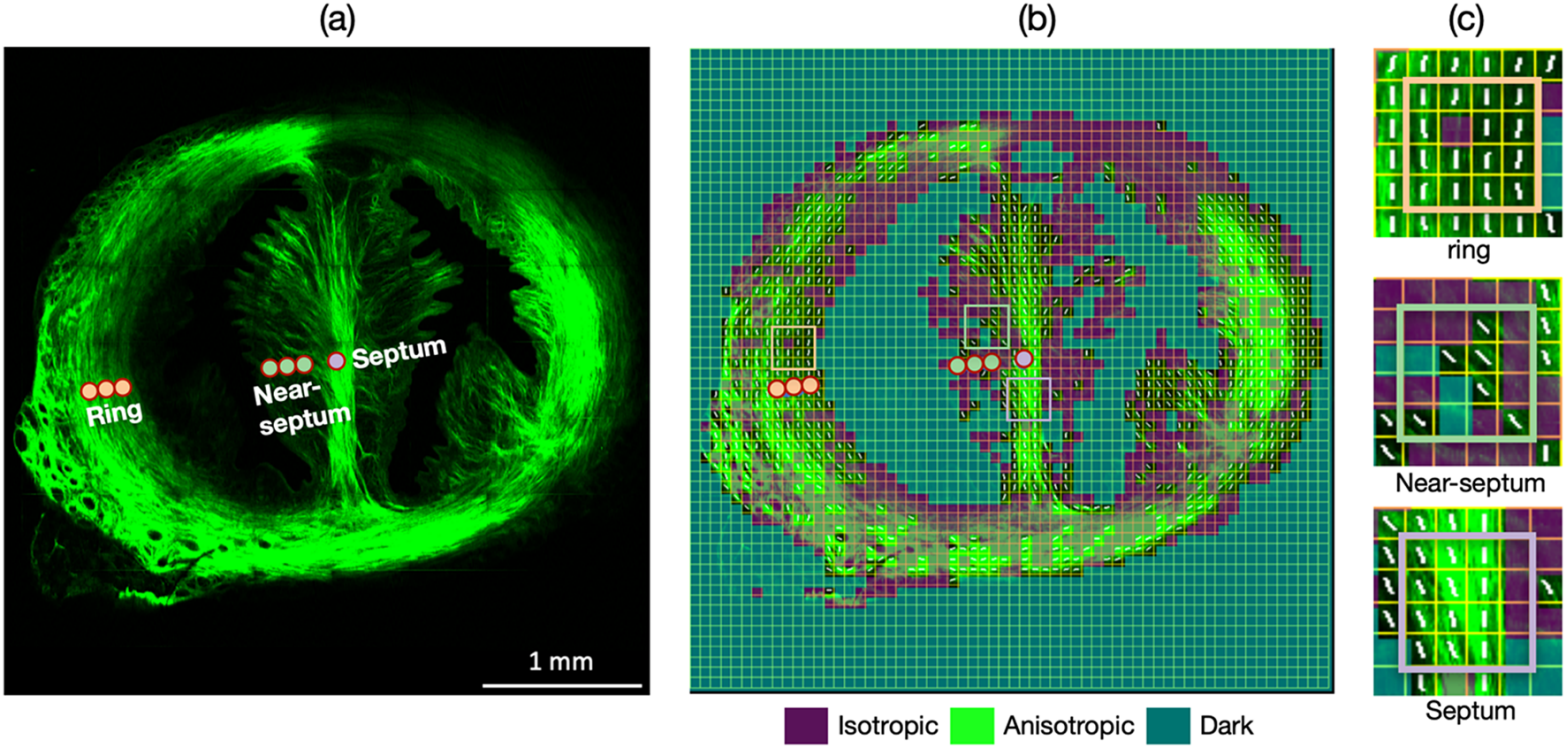
2D FT-SHG analysis of rat cervix tissue. (**a**) 2D SHG image of the rat cervix and (**b**) distribution of isotropic, dark, and anisotropic areas over the SHG image. (**c**) Enlarged images of the ring, near-septum and septum. Colored circles show representative areas that were subsequently captured in 3D. The white lines (**b**,**c**) show the orientation of the anisotropic regions.

Non-pregnant rat cervices were harvested and cryosectioned in preparation for imaging and indentation. An SHG microscope with a 0.25 numerical-aperture (NA) objective lens captured the 2D SHG images of the entire cervical cross section. We separated the cervix into three regions, referred to as the ring, septum, and near-septum, based on their location and distinct structural features (Fig. 1a). The ring region is the high-SHG intensity ring surrounding the two canals. The septum is the bridge connecting the anterior and posterior part of the ring and the near-septum is located between the septum and the canals. Collagen fibers in the ring and septum are preferentially aligned (labeled as anisotropic), while the near-septum includes areas without preferentially aligned fibers (labeled as isotropic). The regions without an SHG signal from collagen fibers are labeled as dark. The use of a low-NA (0.25) objective lens enables a large field-of-view (450 µm) of the tissue with the tradeoff of lower spatial resolution. 2D Fourier transform SHG (FT-SHG) analysis of images from the large fields of view quantitatively identifies the structural features of the cervix and helps to distinguish different regions (Fig. 1b). The details of the 2D FT-SHG method are in our previous work^2^.

**Figure 2:**
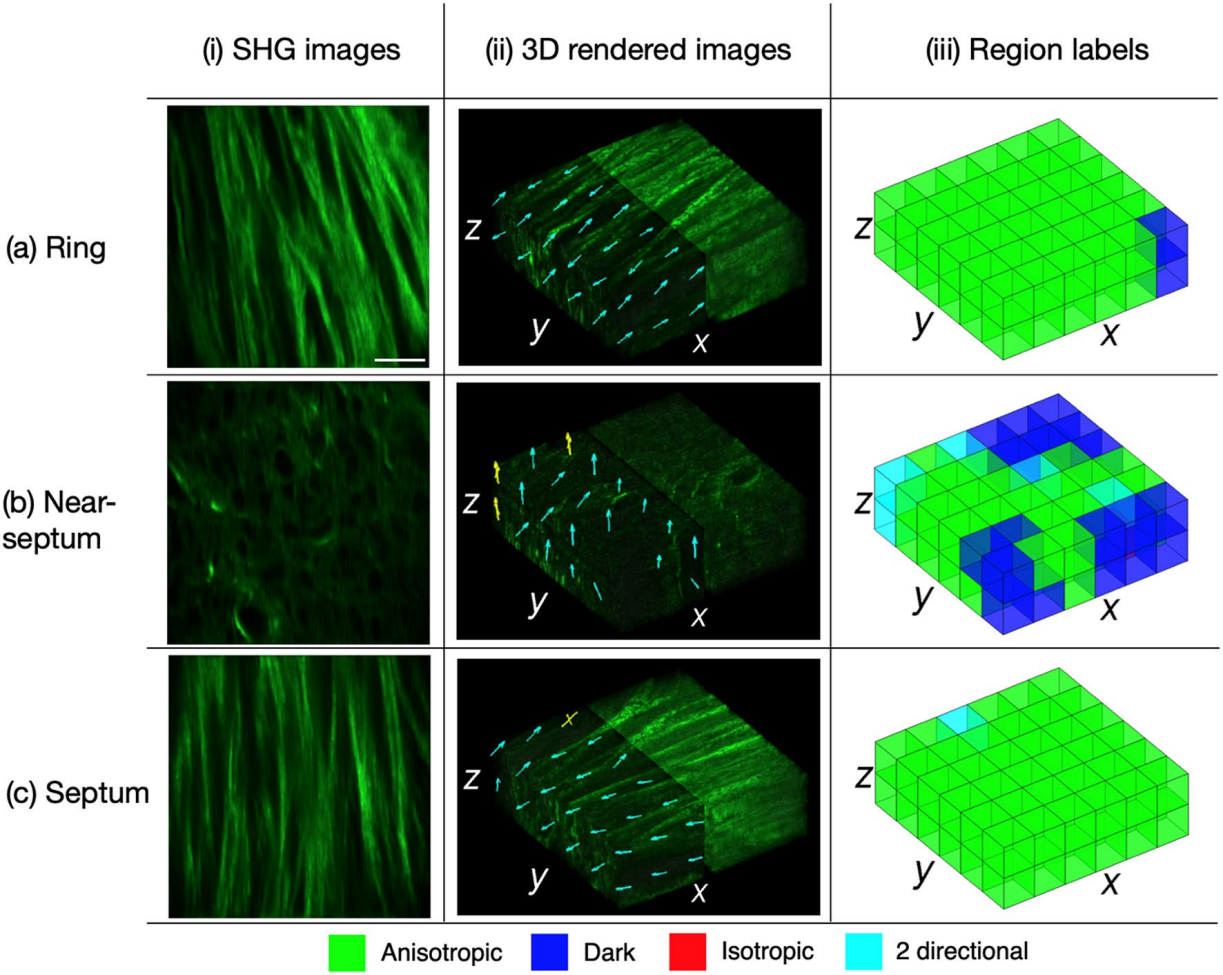
Representative 2D SHG images and results from the 3D FT-SHG analysis of three distinct regions on rat cervix tissue. Each row represents the (**a**) ring, (**b**) near-septum and (**c**) septum. The orientation of the fibers in the anisotropic volume elements are overlaid on the 3D rendered images and shown by arrows [column (ii)]. The cyan and yellow arrows represent volume elements with a single or two preferred fiber orientations, respectively. Each volumetric image (80 × 80 × 13 µm) consists of 72 volume elements (13 × 13 × 13 µm). Scale bar is 15 µm.

A 60× 1.0 NA water immersion objective lens captured the 3D SHG images of the regions of interest. Figure 2 shows representative 2D SHG images and results of the 3D FT-SHG analysis for the ring (Fig. 2a), near-septum (Fig. 2b) and septum (Fig. 2c), respectively. 3D FT-SHG analysis quantifies the fiber orientation and degree of anisotropy in 3D image stacks. Figure 2 column (i) shows representative 2D SHG images; column (ii) shows the 3D rendered images; and column (iii) shows the designated volume elements on the 3D rendered image. The collagen structure in the ring (Fig. 2a) shows highly-aligned fibers as displayed on the 2D SHG image and has in-plane fibers shown on the 3D rendered image. Arrows overlaid on half of the volume on the 3D rendered image indicate the fiber orientation. The region labels in the ring [Fig. 2a column (iii)] indicate that most of the volume elements are anisotropic in terms of fiber orientation. Conversely, the 2D SHG images of the near-septum (Fig. 2b) do not show highly aligned fibers and the overall SHG intensity is lower compared to the ring. The fiber orientation arrows in the 3D SHG image of the near-septum are mostly out-of-plane. Also, there is a higher number of dark and fewer anisotropic volume elements compared to the ring. The septum (Fig. 2c) has similar features as compared to the ring, displaying highly-aligned fibers on the 2D SHG image, having in-plane fibers on the 3D rendered image, and composed of mainly anisotropic volume elements. The yellow arrows in Fig. 2 column (ii) indicate two preferred fiber orientations in a single volume element. For better visualization, the volume elements enlarged in Fig. 2 (13 × 13 × 13 µm), compared with volume elements (6 × 6 × 6 µm) that are processed for calculating the SHG parameters. Criteria for selecting the grid size, or volume element size, are provided in the Methods section.

### SHG image quantification

To adequately represent the microstructure of collagenous tissue and explain its relationship with the mechanical behavior, we quantify the in-plane and out-of-plane dispersion of collagen fibers, in addition to the orientation distribution and density of collagen. The four parameters from the SHG images, including dark, out-of-plane fiber angle (*ϕ*), circular variance (CV), and spherical variance (SV), provide a comprehensive and quantitative representation of the tissue microstructure. The dark parameter is a measure of the volume of the non-collagenous regions of the tissue; *ϕ* is the average angle of the collagen fibers that are out-of-plane relative to the imaging plane where 0° and 90° indicate in-plane and out-of-plane, respectively; CV gives the dispersion of fiber orientations in the imaging plane; and SV is an estimate of the fiber dispersion in 3D. The Methods section offers more details concerning the SHG parameters and the modified CV and SV.

**Figure 3:**
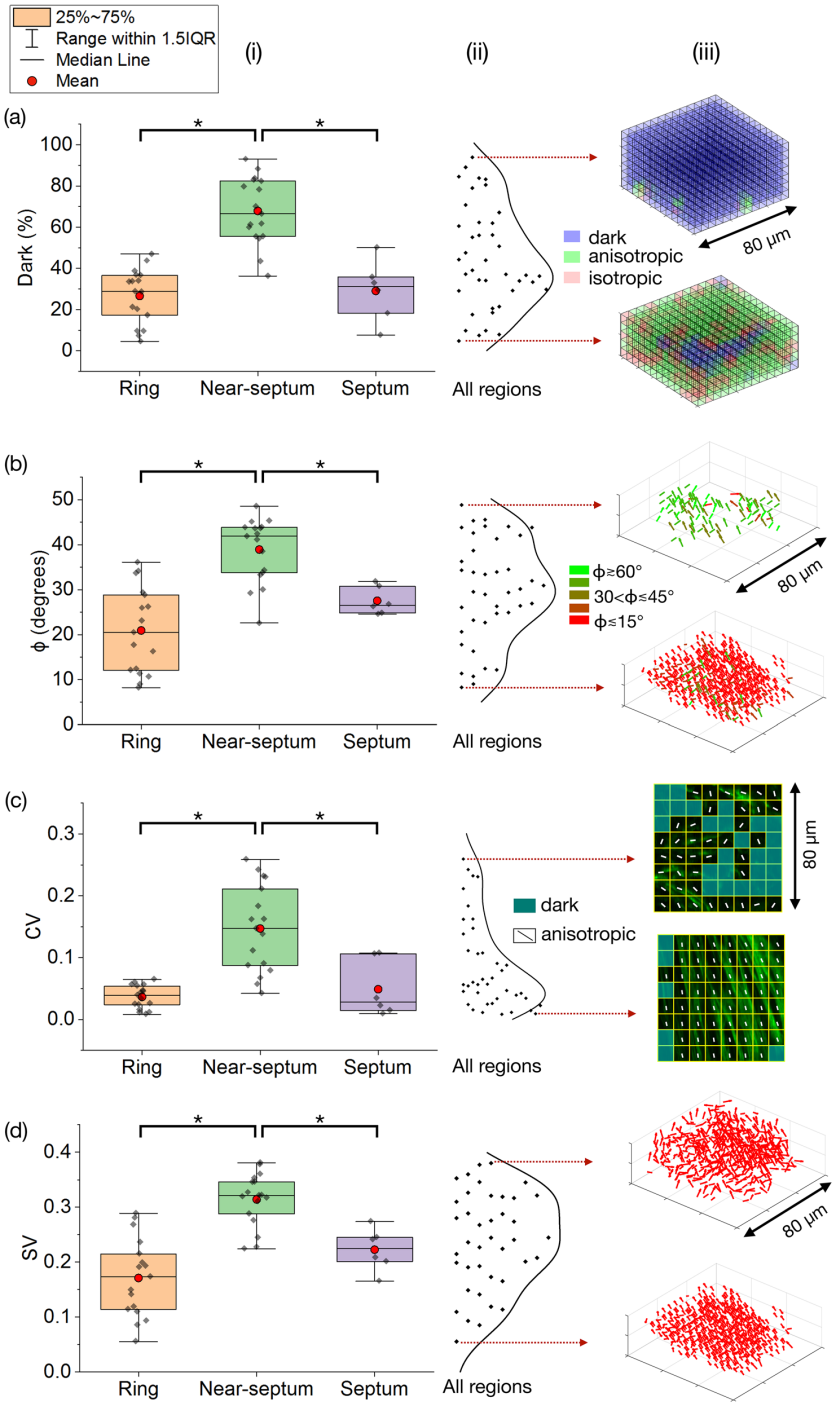
Comparison between the four SHG imaging parameters in the ring, near-septum and septum. The SHG imaging parameters are (**a**) dark, (**b**) out-of-plane fibers, *ϕ*, (**c**) CV, and (**d**) SV. (i) Numerical values in box and whisker plots. (ii) Smooth kernel distribution of the data together from all three regions. (iii) Representative FT-SHG analysis results illustrate the physical meaning of the parameters. Asterisk (*) indicates significant differences (*p* < 0.02).

Figure 3 compares the SHG parameters of different cervical regions of five samples using a one-way ANOVA with post-hoc Tukey analysis. The data are shown using box and whisker plots and the smooth kernel distribution of the data from each of the three regions together are shown in columns (i–ii). Representative results from the 3D FT-SHG analysis shown in column (iii) illustrate the physical representation of the parameters and the range of values in the data set from each of the three regions.

Together, the four SHG parameters quantitatively demonstrate the spatial distribution and organization of the collagen fibers. Here, the near-septum has significantly less collagen and less organized collagen fibers as compared to the ring and septum. The near-septum has a significantly higher percentage of dark volume elements compared with the other two regions (both *p* < 0.02), while the percentage of dark elements in the septum and ring is not statistically different (*p* = 0.933). The higher percentage of dark volume elements in the near-septum indicates a low concentration of collagen and, possibly, the presence of other tissue components. The collagen fibers in the near-septum are on average more out-of-plane, with higher *ϕ* relative to the imaging plane, compared with the ring and septum. Fibers in the ring and septum have smaller *ϕ* and are not significantly different from one another (*p* = 0.197). The higher dispersion of collagen fibers both in-plane (2D) and out-of-plane (3D) in the near-septum is evident; CV and SV are both significantly higher and significantly different in the near-septum compared to the other two regions (both *p* < 0.02). The CV and SV parameters are statistically similar for the ring and septum regions (*p* = 0.863 and 0.166).

### NI and its relationship to microstructural parameters

**Figure 4:**
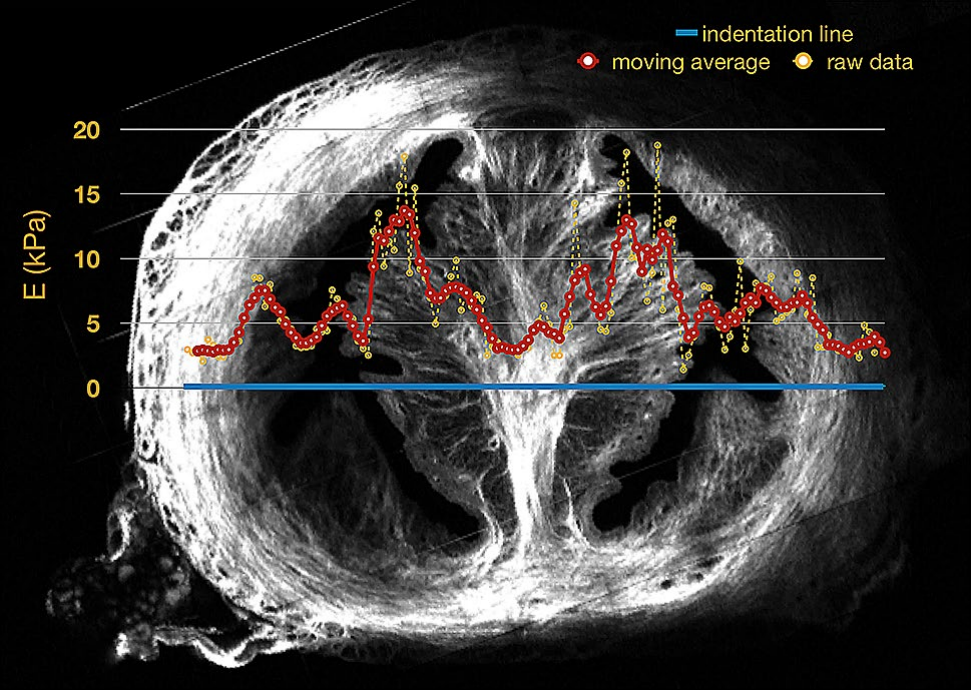
Representative optomechanogram of rat cervix. Raw indentation data (yellow) and the moving average (red, n = 5) of the indentation modulus are aligned with the blue line marking the indentation trajectory.

We performed indentation tests on the same five cervices that we imaged using SHG and calculated the indentation modulus from a general anisotropic contact model^30^ similar to the well-established Hertz model^17^. More details about the indentation tests and the data analysis are in the Methods section. Indenting the sample every 25 µm along the medial–lateral axis, left to right in Fig. 4, reveals the variation of the indentation modulus across the cervix, covering the three regions. The co-registration method aligns the coordinate systems of the indenter and the SHG microscope in order to obtain an optomechanogram (Fig. 4). This visualization represents how the local mechanical properties of the sample are correlated to the collagen organization in that region. Figure 4 explicitly shows the indentation modulus (E) as a function of the probe position, with the indentation trajectory in blue. The indentation modulus changes as a function of tissue microstructure; in particular, the peaks are located in the near-septum.

**Figure 5:**
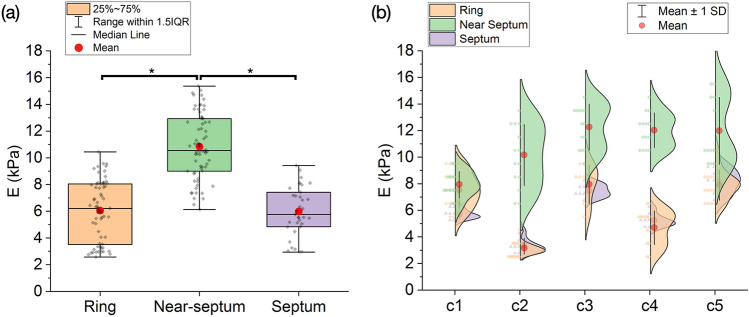
Region-specific indentation modulus of non-pregnant rat cervix. (**a**) Comparison of the indentation modulus in the ring, near-septum and septum. (**b**) Smooth kernel distribution of the indentation modulus in the three regions for five independent cervix samples (c1-c5). Asterisk (*) indicates significant differences (*p* < 0.02).

We segmented the indentation data based on the location in the 2D SHG image of the entire heterogeneous tissue. We compared the indentation modulus of the three cervical regions already identified as the ring, near-septum and septum (Fig. 5a). The indentation modulus is significantly higher in the near-septum (E = 10.84 ± 2.44 kPa; N = 69) compared with the ring (E = 6.053 ± 2.28 kPa; N = 64) and the septum (E = 5.99 ± 1.82 kPa; N = 36) (*p* < 0.02). The indentation modulus of the ring and septum are not significantly different (*p* = 0.990). The mechanical measurements are consistent across the five independent cervix samples (c1-c5, Fig. 5b), demonstrating the repeatability of the experiments.

**Figure 6:**
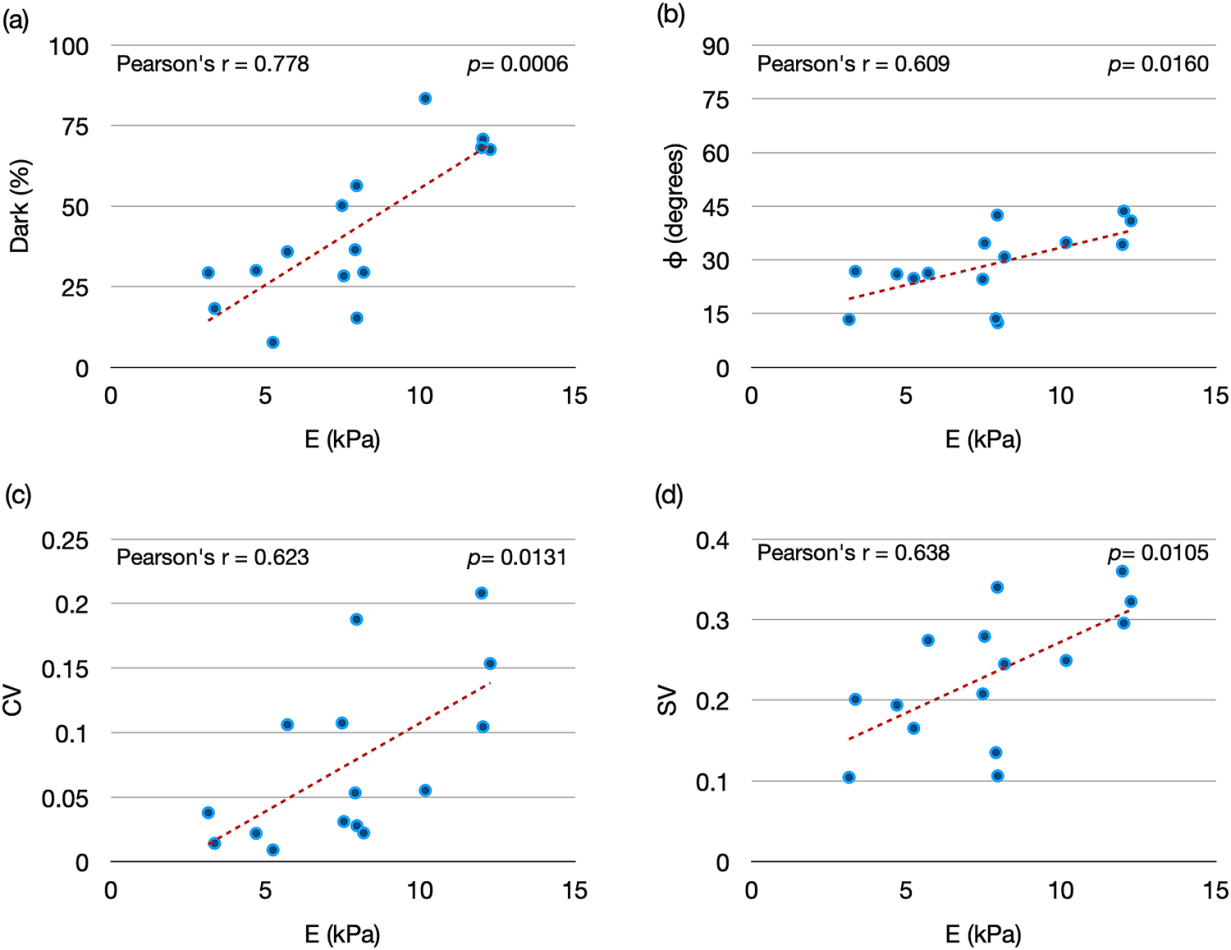
Correlation between the 3D microstructural data and NI results. The indentation modulus has a significant positive correlation with each microstructural parameter. The highest Pearson correlation coefficient was with the dark parameter (0.778), followed by SV (0.638), CV (0.623), and *ϕ* (0.609). The dashed red line shows the linear regression.

We then correlated the different SHG parameters and the indentation modulus. We used the average value of each parameter from the three regions and obtained a total of 15 data points from the 5 cervix samples. Figure 6 shows the correlation between the SHG parameters and E. The Pearson correlation coefficient (Pearson’s r), which quantifies the linear correlation between two parameters, for E and each of the SHG parameters shows that the indentation modulus has the highest correlation with the dark parameter (0.778), followed by SV (0.638), CV (0.623), and *ϕ* (0.609). All of the correlations were significant (*p* < 0.02).

## Discussion

To our knowledge, this study is the first report to quantitatively assess the 3D collagen microstructural and micromechanical spatial distribution of cervical tissue. It also is the first reported example of a combined and co-registered use of qSHG and NI of the same region. Alterations in tissue microstructure and mechanical properties can signal physiological changes and reveal important information about tissue function. In this study, we found region-specific, quantitative microstructural parameters correlate well with the measured indentation modulus in those specific regions. We developed a method to visually and quantitatively describe this correlation, focusing on the role of collagen fiber organization as a determinant of the mechanical properties.

The non-pregnant rat cervix is an example of a less studied, but important heterogeneous tissue. Understanding the microstructure-mechanical properties relationship of the cervix is useful for several reasons. The correlation between quantitative ultrasound (QUS) measures, e.g., acoustic scattering, and microscale properties of the cervix can be identified and quantified to link emerging clinical QUS to other ex vivo measurements. Further, it may help to elucidate the underlying microstructural and micromechanical changes responsible for early collagen remodeling and subsequent sPTB. The data can then be used to develop more accurate diagnostic tools for predicting sPTB. Current clinical screening tools simply do not have the sensitivity to detect risk of preterm birth early enough to implement effective interventions. The current clinical standard is to measure the change in cervical length in high-risk women using clinical ultrasound, but cervical length measurement is most useful for its negative predictive value^31^. There is evidence that changes in tissue microstructure and function may occur before changes in cervical length^29^. It is these microstructural changes, rather than the macrostructural changes, that may have the sensitivity and specificity to signify important new features to detect early cervical remodeling^18, 29^.

A comprehensive and accurate quantitative description of tissue microstructure is important for the analysis of structure–mechanical properties relationship in many tissues. Here, four SHG parameters, dark, *ϕ*, CV, and SV, collectively quantified the collagen fiber organization of the tissue in 2D and 3D. Quantification of the organization in 3D is particularly important for interpretation of the mechanical measurements; 2D imaging techniques are not generally sufficient for acquiring in-depth information about the tissue microstructure. Inaccurate estimation of out-of-plane fiber orientations from 2D images, for example, may lead to overestimation or underestimation of the mechanical properties. We modified the standard definitions of CV and SV before using them for the analysis of the fiber orientation data. The vectors from the FT-SHG analysis represent tissue fibers, which could be equally represented by similar vectors in the opposite direction. Using the directional statistics, e.g., CV and SV, without modifying for this factor would lead to unreliable and inaccurate results.

A previous study^27^ used additional azimuthal angles to transform the axial data (from fiber-like structures) to circular data (vectors) to calculate SV. We formulated the problem differently and used an efficient optimization approach to calculate CV and SV without using a transformation or defining new angles. Another shortcoming of the standard definitions of CV and SV is the lack of proper normalization. The volume elements have different levels of anisotropy, which are not reflected in the standard definitions. We used the magnitude of the FT response from each volume element to assign normalized weights to the orientation data and modified the definitions of CV and SV to reflect the weights in them (see Supplementary Information). In our evaluation of the correlation between mechanical and microstructural properties, the dark, *ϕ*, SV, and CV were all positively correlated with the indentation modulus, with strength of correlation ranging between 0.609 and 0.778. Before modifying the standard definitions, we were not able to identify any significant difference in the SV between the ring and near-septum. Thus, we emphasize the importance of modifying the available mathematical tools for the particular requirements of an image analysis problem.

The SHG parameters, dark, *ϕ*, SV, and CV, discussed in this study can help to improve the structural constitutive models of collagenous tissues, which have been extensively implemented in many studies, e.g.,^32, 33^. Such models have several structural parameters to incorporate the effects of collagen fiber organization in the energy potential. For example, Gasser et al.^32^ introduced a dispersion parameter, κ, in their constitutive model to represent the degree of anisotropy in the fibers. In several studies, e.g.,^34, 35^, the structural parameters are calculated by fitting the models to the data from mechanical tests. However, without an independent measure of the structural organization, the identified material parameters may not be unique. Some studies use techniques such as SHG microscopy^36^ and staining^37^ to directly determine the structural parameters independent from the mechanical tests. These studies, however, do not fit the constitutive models to the local mechanical response of the tissue, and consequently, the relationship between the structural and mechanical properties at the microscale remains ambiguous. Fitting the models to the micromechanical response of the tissue after finding the co-registered microstructural parameters from independent imaging can help to improve the accuracy of the models. Multi-scale and subject-specific finite element models can be developed based on these co-registered measurements to better predict the elastic behavior of the tissue.

The indentation modulus was significantly higher in the near-septum compared with the two other regions. The SHG image data, however, showed a lower concentration of collagen in the near-septum, where the collagen fibers were less organized and fiber orientations were more out-of-plane. This observation was unexpected, but was consistent across experiments on multiple independent cervices. We anticipated a higher modulus from the areas dense with collagen fibers, but our results suggest that collagen concentration and orientation are not the only factors affecting the mechanical properties for this mode of deformation. For example, GAGs support tissue in compression^38^ and their higher concentration in the near-septum^39^ could potentially explain the higher indentation modulus of this region. In contrast, the ring and septum had similar mechanical and structural properties. We also note that the low SHG signal does not always indicate that there is a low concentration of collagen fibers. The SHG signal is extremely weak or vanishing once the fibers are aligned out of the image plane and significantly drops when the fibers are perpendicular to the image plane^40^. Thus, the low SHG intensity of near-septum could indicate the majority of fibers are out-of-plane and this is proved by the 3D orientation calculation shown in Fig. 3. The spatial variations of properties across the cervical regions affect the overall response of the tissue to physiological loads. Thus, it is important to include these local variations in finite element models of tissue using a higher resolution material properties assignment, based on the anatomical site and patient-specific images of collagen fiber organization, to accurately model the complex mechanical behavior of the cervix and any other heterogeneous tissue.

We used the indentation modulus instead of the Young’s modulus to avoid imposing any restrictive assumptions on the elastic anisotropy of tissue. The indentation modulus is a more general measure of the mechanical response and can be used for both isotropic and anisotropic materials. It is a function of all of the components of the elasticity tensor, material orientation, and indentation orientation^30^. For isotropic materials, the general anisotropic formulation^30^ reduces to the Hertz model^17^ and the indentation modulus is sufficient to calculate the Young’s modulus when the Poisson’s ratio is known or assumed. For anisotropic materials like cervical tissue, however, the indentation modulus is not enough to fully characterize the material. Often, complementary bulk tests, complicated data analysis, and specialized equipment are also required for the characterization, e.g.,^41^. Our previous work^42^ presented a simple indentation-based approach for the characterization of soft anisotropic tissues, but it was limited to mm-scale indenters, and thus, unsuitable for microscale characterization. While the indentation modulus provides a useful estimate of the mechanical properties, anisotropic characterization at microscale may provide further insights into the tissue microstructure and micromechanics. For example, each component of the elasticity tensor could have a different spatial distribution, change differently with gestational age, or correlate with different SHG parameters. Therefore, improved techniques for anisotropic, mechanical characterization of tissue at microscale could help to explain tissue remodeling, mechanobiology, and function in more detail.

We used the cervix as a test case to develop our methodology. The optomechanogram, however, can be useful in many other areas of research such as studying the cornea and the tumor microenvironment. In the former case, the optomechanogram could reveal the correlation between the spatially varying tissue microstructure and biomechanical changes of the healthy and diseased cornea and pave the way for identifying new treatment targets and early diagnosis pathways. For the tumor microenvironment, an optomechanogram for cancerous tissue could help elucidate the role of mechanical signals in cancer development.

There were several limitations to this study. First, the accuracy of the optomechanogram depended on the spatial accuracy of the co-registration to approximately the size of the probe radius, ~ 50 µm. Semi-manual stitching of the images, among other factors, reduced the accuracy of locating the indentation points in the SHG images. This source of error can be eliminated by using advanced commercialized software. Another limitation was the number of 3D images from the different regions. We captured a maximum of three volumetric 3D images (80 × 80 × 26 µm from each region per cervix. Imaging more regions could allow us to refine the optomechanogram and investigate the structural properties of more areas of the sample. However, with the data presented we had consistency across five cervices and demonstrated significant differences across regions. Finally, we did not measure permeability, diffusivity, and visco or poroelastic properties. These properties would be useful for more in-depth structure–function relationship studies, but such an in-depth study of this kind was not possible with the current experimental set-up and we only measured the indentation modulus as an important indicator of the mechanical properties. In future work, we will characterize the micromechanical and microstructural properties of the rat cervix at different gestational ages to further understand the structure–function relationship and microenvironment of the tissue and pursue other collagenous tissues.

## Conclusion

In this study, we characterized the 3D collagen microstructure and micromechanical properties of a complex collagenous tissue and, for the first time, reported the correlation between them using a custom co-registration method. 3D qSHG imaging provided the microstructural information and NI measured the local indentation modulus. We characterized rat cervix as an example of a complex and heterogeneous tissue and introduced the optomechanogram to visually illustrate and quantify the direct link between the tissue microstructure and micromechanical properties. We found that the indentation modulus is significantly correlated with the qSHG parameters describing the collagen concentration, disorganization in collagen fiber orientation, and the degree of out-of-plane collagen-fiber alignment. The SHG parameters, and their correlation with micromechanical properties, can improve the constitutive models of the cervix and other collagenous tissues by providing the local structural parameters. This work contributes to understanding the 3D collagen microstructure and its relation to mechanical function of the tissue, which could lead to improved detection methods for diseases and conditions, such as sPTB. Since both the optical and mechanical techniques can be applied to other tissues, the proposed method can be extended to any collagenous tissue. Moreover, our framework for co-registration of optical and mechanical data, and the resulting optomechanogram, is applicable to other 3D imaging platforms used in conjunction with NI, thereby permitting general optomechanical metrology of materials.

## Materials and methods

### Sample preparation

All procedures were approved by the Institutional Animal Care and Use Committee at the University of Illinois at Urbana-Champaign. All protocols were performed in accordance with the relevant guidelines and regulations. Five 12-week-old non-pregnant rats (Sprague Dawley’s) were euthanized and the cervical tissue was immediately harvested and stored at − 80 °C. The tissue was thawed at room temperature for 10 min and the surrounding tissue was removed. Samples were embedded in optimal cutting temperature compound, with two cuboids made of birch craft wood (4 × 2 × 2 mm) located 1–2 mm from the tissue, and cryosectioned at − 20 °C (Leica Biosystems, Wetzlar, Germany) 3 mm above the external orifice, perpendicular to the cervical canals. We sectioned the tissue before NI to reduce the surface roughness and expose the area of interest (mid cervix). Surface roughness can significantly influence the measurement of indentation modulus and cryosectioning is an accepted method for soft tissues to minimize this error^43, 44^. The frozen cervix tissue and birch cuboids were glued to a microscope slide using cyanoacrylate. The tissue was washed with buffer solution after the adhesive cured. The birch cuboids had two roles: they physically supported the tissue during imaging to prevent tissue deformation and served as fiducial references for co-registration.

### NI

A Piuma nanoindenter (Optics11, Amsterdam, Netherlands) acquired the force-indentation data from which the indentation modulus was derived using a general anisotropic contact model^30^. The spherical probe radius was 58 µm and the cantilever stiffness was 0.49 N/m. The built-in camera of the nanoindenter captured brightfield images of the entire cervix cross-section and fiducial references for co-registration, described below. To locate the indentation points on the image of cervix cross-section, another set of brightfield images were captured right before indentation, when the probe was in contact with the sample. The sample was indented every 25 µm along two medial–lateral lines 25 µm apart. The maximum indentation depth was 5 µm, with a displacement rate of 5 µm/s. The samples were submerged in buffer solution throughout indentation.

### SHG imaging

A custom SHG microscope imaged the cervix samples. A Ti:Sapphire laser illuminated the samples producing 100-fs centered at 780 nm. A 10× 0.25 NA objective lens on an inverted microscope (Olympus, Tokyo, Japan) collected the SHG signal for the 2D images. To construct an image of the whole cross-section of the cervix, a motorized stage scanned the field of view (535 μm × 535 μm) with a step size of 500 μm and the acquired images were stitched together. In addition to the SHG images, a set of brightfield images of the whole cross-section were acquired for co-registration. 3D image stacks were obtained in three locations in the ring, three locations in the near-septum, and one location in the septum. We used a 60× 1.0 NA water immersion objective lens to collect the 3D-SHG images and the z step size was 350 nm. The samples were placed on a No. 1.5 cover glass-bottom dish (MatTek, Ashland, MA) with buffer solution to remain hydrated during imaging.

### SHG parameters

The SHG parameters including dark volume, out-of-plane fiber angle (*ϕ*), CV, and SV were computed for each sample using the orientation data provided by FT-SHG analysis^12^. The dark parameter indicates the non-collagenous volumes of the sample and is defined based on the intensity of the SHG signal in the image stack. The volume elements (6 × 6 × 6 µm) were labeled as dark if the average intensity was below a fixed threshold (0.1). The percentage of the dark volume elements over the entire volume was defined as the dark parameter. The *ϕ* parameter represents the average angle of the collagen fibers with respect to the x–y imaging plane, where 0° and 90° indicate in-plane and out-of-plane, respectively. *ϕ* is average of the out-of-plane fiber angle from all of the volume elements over the volume of interest. CV is the dispersion of the fiber orientations in the imaging plane with a value between 0 (unidirectional orientation) and 1 (uniformly dispersed). Similarly, SV describes the dispersion of fiber orientations in 3D, taking into account both in-plane and out-of-plane orientations. Both CV and SV consider the orientations of all the volume elements in the region of interest. The resolution of the orientation data, determined by the size of the volume elements, was chosen based on several factors such as collagen density, size of the collagen fibers, and crimping patterns to ensure accuracy.

### Co-registration of the NI and SHG data

The indentation points were located on the SHG images using a custom co-registration method, illustrated in Fig. S1. Bright-field images of the cervix cross-section with the two supporting birch cuboids were taken in both systems to facilitate the co-registration. The cuboids and the tissue boundaries served as fiducial references. The indentation positions were determined using the images from the nanoindenter in which the probe was in contact with the sample. The bright-field images from the nanoindenter and the SHG microscope were co-registered semi-manually in ImageJ 1.52 (National Institute of Health, Bethesda, MD) using the fiducial references. Finally, the indentation points were overlaid on the SHG image using the coordinates taken from the two sets of bright-field images. The co-registration error was measured by using microscope slide grids (Thomas Scientific, Swedesboro, NJ) and comparing the two bright-field images obtained by the nanoindenter and SHG microscope. The amount of mismatch between the two bright-field images was ~ 50 μm for a 6 × 6 mm region.

### Statistical analysis

The Origin 9.6 software package (OriginLab Corporation, Northampton, MA) was used for the statistical analysis. A one-way ANOVA determined the difference between the means of different regions, namely the ring, near-septum, and septum. A posthoc Tukey analysis indicated which groups differed from one another following the one-way ANOVA. Calculated Pearson correlation coefficients demonstrated the linear dependence of the indentation modulus and each of the four SHG parameters. The significance level is set at 0.02 and all the calculated *p* values are included in the Supplementary Information.

### Modified definition of SV and CV

The process of calculating the SV from the orientation data is described in this section. The CV, as a special case of the SV for in-plane vectors, can also be calculated from this general formulation. The standard SV of a set of vectors, $\hat{x}_{i}$, with direction cosines $\left[ {l_{i} ,m_{i} ,n_{i} } \right]$ ($i = 1,..,n$), is calculated as follows^45^:

1 $$\hat{R} = \left( {\mathop \sum \limits_{i = 1}^{n} l_{i}^{2} + \mathop \sum \limits_{i = 1}^{n} m_{i}^{2} + \mathop \sum \limits_{i = 1}^{n} n_{i}^{2} } \right)^{0.5} ,$$

2 $$SV = \frac{{n - \hat{R}}}{n} .$$

If the vectors represent the collagen fibers in tissue, another set of vectors with the opposite directions relative to the given vectors, i.e. $- \hat{x}_{i}$, can equally represent the same fibers. Also, the degree of anisotropy of each volume element, quantified by a set of normalized weights, should be reflected in the SV. Thus, the following modified definition is used for the calculation of SV. For a set of vectors $\hat{x}_{i}$, with direction cosines $\left[ {l_{i} ,m_{i} ,n_{i} } \right]$ ($i = 1,..,n$), and normalized weights, $wn_{i}$, the input space is defined as a set of $2n$ vectors, $\hat{X}_{i}$, which consists of $\hat{x}_{i}$ and $- \hat{x}_{i}$. The modified SV is calculated as follows

3 $$\hat{R}_{1} = wn_{m} \hat{X}_{m} { }\quad where\,\,m = \arg \mathop {\max }\limits_{i} \frac{1}{n}\mathop \sum \limits_{j = 1}^{n} wn_{j} cos(A_{ij} ) ,$$

4 $$\hat{R}_{i + 1} = \hat{R}_{i} + wn_{m} \hat{X}_{m} \quad where\,\,m = \arg \mathop {\max }\limits_{i} { }\left( {wn_{j} \cos \left( {\tan^{ - 1} \frac{{\hat{R}_{i} \times \hat{X}_{j} }}{{\hat{R}_{i} \cdot \hat{X}_{j} }}} \right)} \right) ,$$

5 $$SV = 1 - \hat{R}_{n} ,$$

where $A_{ij}$ is the angle between the *i*th and *j*th members of the input space. A similar formulation can be used for CV. For every input vector, $\hat{x}_{i}$, $n_{i}$ is set to zero, since $\hat{x}_{i} \in R^{2}$. Other steps are similar to the steps needed for calculating the SV. More details are provided in the Supplementary Information.

## Supplementary information

Supplementary information.

## Data Availability

The data that support the findings and plots of this study are available from the corresponding authors upon reasonable request.
