# Supplementary material for: An optomechanogram for assessment of the structural and mechanical properties of tissues
Source: Sci Rep. 2021 Jan 11;11:324. doi: 10.1038/s41598-020-79602-6 (PMC7801423; doi:10.1038/s41598-020-79602-6)

**Title : An optomechanogram for assessment of the structural and mechanical properties of tissues**

**Author list : W. Lee^1†^, A. Ostadi Moghaddam^2†^, S. Shen^3^, H. Phillips^4^, B. L. McFarlin^5^, A. J. Wagoner Johnson^2,6,7*^, K. C. Toussaint, Jr.^8*^**

†Co-first authors; ^*^Co-corresponding authors. Email: [ajwj@illinois.edu](mailto:ajwj@illinois.edu), [kimani_toussaint@brown.edu](mailto:kimani_toussaint@brown.edu)

*^1^Department of Mechanical Engineering, University of Colorado at Boulder, Boulder, CO 80309, USA.*

*^2^Department of Mechanical Science and Engineering, University of Illinois at Urbana-Champaign, Champaign, IL 61820, USA.*

***^3^****Center for Health, Aging, & Disability (CHAD), College of Applied Health Science, University of Illinois at Urbana-Champaign, Champaign, IL 61820, USA.*

***^4^****Department of Veterinary Clinical Medicine, University of Illinois at Urbana-Champaign, Urbana, IL 61801, USA.*

***^5^****Department of Women, Children and Family Health Science, University of Illinois College of Nursing, Chicago, IL 60612, USA.*

***^6^****Carle Illinois College of Medicine, University of Illinois at Urbana-Champaign, Champaign, IL 61820, USA.*

***^7^****Carl R. Woese Institute for Genomic Biology, University of Illinois at Urbana-Champaign, Urbana, IL 61801, USA.*

***^8^****School of Engineering, Brown University, Providence, RI 02912, USA.*

## **Supplementary Information**


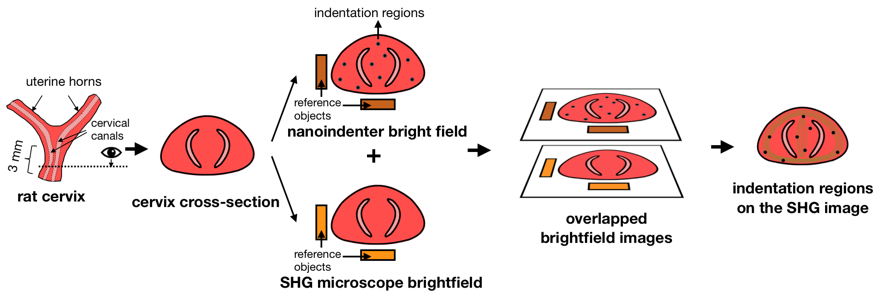


**Figure S1.** Illustration of the co-registration method between the nanoindenter and SHG microscope coordinate systems.

**Calculation of SV and CV for fibrous materials**

Quantifying the dispersion of collagen fibers in 2D and 3D helps to evaluate the tissue condition and provides a tool for understanding the relationship between the tissue structure and properties. The SV, as a measure of dispersion between a set of vectors in 3D, is a candidate for quantifying the collagen fiber dispersion from SHG . The FT analysis gives a set of vectors that represents the local fiber orientation of a volumetric SHG image. However, we need to address two issues before using the SV for quantifying tissue images. First, a set of vectors with opposite directions relative to the vectors from the FT analysis represents the same fibers. Choosing the representative vectors from the two opposing groups arbitrarily leads to inconsistent and inaccurate calculation of the SV. Second, we have to set an anisotropy threshold for the orientation analysis and neglect the orientation of the volume elements that fall below the threshold. Every region above the threshold is included in SV, regardless of the anisotropy level. We propose a modified definition for SV to address both issues.

Consider a set of $n$ vectors,$\hat{x}_{i}$, with direction cosines $[l_{i},m_{i},n_{i}]$ ($i=1,..,n$), that represents the orientation of fibers within a volumetric image. For each vector, we can use the FT response to calculate a weight, $w_{i}$, proportional to the anisotropy level. The following steps are required for calculation of SV:

1. Normalize the weights. The sum of the normalized weights should be equal to one for each volumetric image.

${wn}_{i}=\frac{w_{i}}{\sum_{i=1}^{n} w_{i}}$ (1)

Where $wn_{i}$ represents the normalized weight of the $i^{th}$ vector.

2. Calculate the input space $\hat{X}_{i}$. The input space includes the representative vectors$,\hat{x}_{i},$ from the FT analysis and a set of corresponding vectors, ${-\hat{x}}_{i}$, that equally represents the fiber orientation of the volume elements in the same image

$\hat{X}_{i}=\left[ \begin{matrix} l_{i} & m_{i} & n_{i} \\ -l_{i} & -m_{i} & -n_{i} \end{matrix} \right]$ (2)

For each member of the input space, we define its corresponding admissible vector space as a set of $n-1$ vectors that gives the maximum resultant vector.

3. Calculate the relative angle matrix ($A_{ij})$ for the input space. $A_{ij}$ gives the angle between two members of the input space $\hat{X}_{i}$ and $\hat{X}_{j}$

$A_{\mathrm{ij}}=\tan^{-1} \frac{\left\| \hat{X}_{i}\times\hat{X}_{j} \right\|}{\hat{X}_{i}.\hat{X}_{j}}$ (3)

4. Initiate the resultant vector from the $m^{th}$ component of the input space, which is closest, on average, to every other member of its corresponding admissible vector space

$m=\arg\max_{i} \frac{1}{n}\sum_{j=1}^{n} {\mathrm{wn}_{j}cos(A}_{\mathrm{ij}})$ (4)

$\hat{R}_{1}={\mathrm{wn}_{m}\hat{X}}_{m}$ (5)

Where $j=1,2,..n$ are members of the admissible vector space for $\hat{X}_{i}$ and $\hat{R}_{1}$ is the first iteration of the resultant vector.

5. Iterate $(n-1)$ times to find the maximum resultant vector within the input space

$m=\arg\max_{i} \left( {\mathrm{wn}_{j}cos(tan}^{-1} \frac{\left\| \hat{R}_{i}\times\hat{X}_{j} \right\|}{\hat{R}_{i}.\hat{X}_{j}}) \right)$ (6)

$\hat{R}_{i+1}=\hat{R_{i}}+{\mathrm{wn}_{m}\hat{X}}_{m}$ (7)

6. Calculate the SV using the $n^{th}$ iteration of the resultant vector

$SV=1-\left\| \hat{R}_{n} \right\|$ (8)

A similar formulation can be used for CV. For every input vector$,\hat{x}_{i}$, $n_{i}=0$ since $\hat{x}_{i}\in R^{2}$. Other steps are similar to the steps needed for calculating the SV.

**Table S1.** Calculated *p* values of the near-septum, ring and septum region with respect to the qSHG and NI parameters.


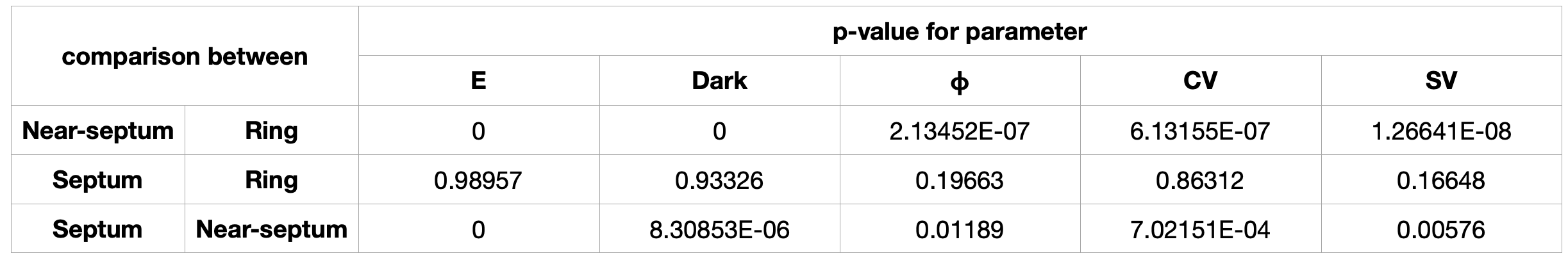

Supplement: Supplementary file 1 — Supplementary information. [file 41598_2020_79602_MOESM1_ESM.docx]
